# Predicting in-hospital mortality and unanticipated admissions to the intensive care unit using routinely collected blood tests and vital signs: Development and validation of a multivariable model^☆^

**DOI:** 10.1016/j.resuscitation.2018.09.021

**Published:** 2018-12

**Authors:** Oliver C. Redfern, Marco A.F. Pimentel, David Prytherch, Paul Meredith, David A. Clifton, Lionel Tarassenko, Gary B. Smith, Peter J. Watkinson

**Affiliations:** a Centre for Healthcare Modelling and Informatics, University of Portsmouth, Portsmouth, UK; b Institute of Biomedical Engineering, Department of Engineering Science, University of Oxford, Oxford, UK; c Research and Innovation Department, Portsmouth Hospitals NHS Trust, Portsmouth, UK; d Faculty of Health and Social Sciences, Bournemouth University, Bournemouth, UK; e Nuffield Department of Clinical Neurosciences, Oxford University Hospitals NHS Trust, Oxford, UK

**Keywords:** Physiological monitoring, Early warning score, Vital signs, Laboratory test results

## Abstract

**Aim:**

The National Early Warning System (NEWS) is based on vital signs; the Laboratory Decision Tree Early Warning Score (LDT-EWS) on laboratory test results. We aimed to develop and validate a new EWS (the LDTEWS:NEWS risk index) by combining the two and evaluating the discrimination of the primary outcome of unanticipated intensive care unit (ICU) admission or in-hospital mortality, within 24 h.

**Methods:**

We studied emergency medical admissions, aged 16 years or over, admitted to Oxford University Hospitals (OUH) and Portsmouth Hospitals (PH). Each admission had vital signs and laboratory tests measured within their hospital stay. We combined LDT-EWS and NEWS values using a linear time-decay weighting function imposed on the most recent blood tests. The LDTEWS:NEWS risk index was developed using data from 5 years of admissions to PH, and validated on a year of data from both PH and OUH. We tested the risk index’s ability to discriminate the primary outcome using the c-statistic.

**Results:**

The development cohort contained 97,933 admissions (median age = 73 years) of which 4723 (4.8%) resulted inhospital death and 1078 (1.1%) in unanticipated ICU admission. We validated the risk index using data from PH (n = 21,028) and OUH (n = 16,383). The risk index showed a higher discrimination in the validation sets (c-statistic value (95% CI)) (PH, 0.901 (0.898–0.905); OUH, 0.916 (0.911–0.921)), than NEWS alone (PH, 0.877 (0.873–0.882); OUH, 0.898 (0.893–0.904)).

**Conclusions:**

The LDTEWS:NEWS risk index increases the ability to identify patients at risk of deterioration, compared to NEWS alone.

## Background

Multiple audits of acute hospitals in the UK have concluded that inadequate monitoring of patients and a failure to identify their deterioration contribute to avoidable harm [1,2]. The routine use of an early warning score (EWS) is recommended to identify those patients most at risk of major adverse outcomes, such as death, cardiac arrest and unanticipated transfer to the intensive care unit (ICU) [[3], [4], [5], [6]]. Early warning scores provide a simple composite measure of the extent of physiological abnormality, usually based on vital signs (e.g. heart rate, blood pressure, respiratory rate). In addition, they are easily interpretable by clinical staff and can be calculated either manually or by electronic systems [4,7,8]. Although measured less often than vital signs, several laboratory test results have also been shown to be predictive of adverse outcomes [[9], [10], [11], [12], [13]]. We have previously described the development of an EWS using blood tests routinely performed on admission to hospital [14]. Furthermore, work involving our group [15] and others [16,17] has also shown how combining blood tests and vital signs measurements can increase the ability of model scoring systems to detect high-risk patients.

In this paper, we hypothesise that combining the information from laboratory tests and vital signs can be used to identify patients at high risk of death and ICU admission in the short term (for example, 24 h). We report the development of a novel scoring system, the LDTEWS:NEWS *risk index*, that combines one based on routinely collected vital signs (the National Early Warning Score, NEWS) [3,4] and another based exclusively on laboratory tests (the Laboratory-Decision Tree Early Warning Score, LDT-EWS) [14]. In order to simulate “real-world” use, the risk index is calculated from patients’ most recent results, as would be displayed on the hospital system. To be comparable to the original NEWS and LDT-EWS publications [3,14] and increase case-mix homogeneity in hospital, we validate the risk index using emergency medical admissions. We assess its ability to predict in-hospital mortality and unanticipated transfer to the ICU from the general wards in four acute hospitals.

## Materials and methods

This study is reported in line with the TRIPOD statement [18].

### Ethical approval

Health Research Authority approval was obtained for gathering the data used in this study from the Research Ethics Committee (REC reference: 16/SC/0264 and 08/02/1394).

### Source of data

This was a retrospective cohort study of routinely collected electronic data as part of the Hospital Alerting Via Electronic Noticeboard (HAVEN) project [19], which includes admissions to the Oxford University Hospitals (OUH) (from January 2016 to December 2016) or to Portsmouth Hospitals (PH) (from January 2011 to December 2016). The following administrative and clinical data are included in the database: electronic recordings of vital signs, laboratory test results, patient demographics and information related to occurrences and timings of (in-hospital) patient death and unanticipated ICU admissions as identified from the hospitals’ clinical information systems. Vital sign observations in all hospitals were recorded electronically at the bedside using VitalPAC™ (System C Healthcare, www.systemc.com) in Portsmouth [8] and the System for Electronic Notification and Documentation (SEND, Drayson Technologies, www.draysontechnologies.com) in Oxford [7].

### Participants and sample size

Admissions to four acute hospitals in the UK were considered in this study: the Queen Alexandra Hospital (a large district hospital) as part of the PH group; and the John Radcliffe Hospital (a large university hospital), the Horton General Hospital (a small district hospital) and the Churchill Hospital (a large university cancer centre), which are all within the OUH group. For Portsmouth hospitals, these data comprised completed admissions from January 2012 to December 2016; for Oxford hospitals from January 2016 to December 2016. Only adult (at least 16 years of age) admissions where the discharge status of the patient episode was recorded are included in the database. All hospital admissions where patients were admitted as an emergency [20] to a subset of higher-risk medical specialties (see [21]), and where patients had, at least, one recorded set of vital signs sufficient to calculate a NEWS score, were considered for inclusion in the analysis. This is in keeping with the cohorts on which LDTEWS and NEWS were developed [4,14]. We excluded admissions where a) the patient was discharged alive from hospital before midnight on the day of admission, and b) no vital-sign observations were recorded in the 24 h prior to the first day of ICU admission, discharge or death (as a proxy for patients who were likely to be on end-of-life pathways, as this information was not available).

### Outcomes

The primary outcome was the first of in-hospital death or unanticipated admission to ICU within 24 h of a given set of vital-sign observations [21]. We have also considered, as secondary outcomes, each individual adverse event separately: in-hospital death within 24 h, and unanticipated ICU admission within 24 h.

### Predictors

All vital signs and laboratory test results (and corresponding timings) required to calculate NEWS and LDTEWS (14 variables in total) were extracted for each admission. Each set of vital signs include heart rate, systolic blood pressure, respiratory rate, body temperature, a neurological status assessment using either the Alert-Verbal-Painful-Unresponsive (AVPU) scale or the Glasgow Coma Scale (GCS), peripheral oxygen saturation from pulse oximetry (SpO_2_), a record of whether the patient was receiving supplementary oxygen at the time of SpO_2_ measurement, and the date and time of the observation. Where neurological status had been assessed using the GCS, we converted the GCS value to the AVPU scale, as previously described [22]. An aggregate NEWS score was calculated from each set of vital signs using the corresponding weightings [4] (see Table 1 for the weightings of NEWS and LDTEWS).

**Table 1:** NEWS and LDTEWS (for both males and females) cut-offs and weightings.

Laboratory serum blood test results and their recorded times (at which they were received by the laboratory) were obtained for the following: albumin (g/L), creatinine (μmol/L), haemoglobin (g/L), potassium (mmol/L), sodium (mmol/L), urea (mmol/L) and white cell count (WCC) (10^9^ cells/L). These specific blood tests were originally chosen for the development of the LDTEWS score as they are routinely measured in the majority of patients admitted as an emergency to medical specialties [14]. Laboratory test results were linked to individual admissions if specimens were received by the laboratory between the day prior to admission and the day of discharge – extending to the day prior to admission was to account for patients admitted through the emergency department overnight.

### Missing data

As it is rare for laboratory variables to be measured at the same time as vital signs, the model must allow for missing data. We addressed this by using the most recent value of each variable (vital sign or laboratory test result) when computing the risk score, limiting the acceptable time that a measurement can be carried forward (e.g. to 5 days). If a variable is completely missing for a particular admission, a score of zero is assigned to the EWS component of that variable.

### Development and validation sets

After exclusion criteria were applied, we split the database into development and validation sets by time, such that (1) we developed a model using data from one period and evaluated its performance using data from a distinct, more recent period, and (2) we also performed an external validation of the model using data from a different organisation [18]. The development dataset comprises all admissions from January 2011 and December 2015 to the PH group. Two validation sets were extracted from the remaining admissions: one dataset that includes all admissions to PH between January 2016 and December 2016, and one validation set that includes all admissions to the OUH group that took place between January 2016 and December 2016.

### Statistical analysis methods

To develop the risk model, our approach assumes that there is a single number that characterises the patient’s current condition at the time that a vital-sign observation set is recorded. Hence, for each admission, a set of linked vital signs and laboratory test results is generated by carrying forward the most recent value of each of the 7 laboratory tests with each vital sign observation set. All vital signs and laboratory test results are then assigned weights according to NEWS and LDTEWS (see Table 1), and the sum of the corresponding weights results in the two aggregate scores. In order to have both summary scores on a common scale (0–1), we divided NEWS and LDTEWS by their respective possible maximum values (20 and 15, respectively).

The final score, the LDTEWS:NEWS *risk index*, was determined using a weighted sum of the two scores, NEWS and LDTEWS, calculated at a given time. Laboratory tests are usually performed less frequently than vital-sign observations. Variations in the frequency of blood sample collection, the lag between measurement times and the time the results are available in the electronic system lead to asynchronous data input into risk index computation. We accommodated this by using the most recently available laboratory result to recalculate the risk index at each new observation of vital signs. As these test results become less “current”, their relevance to the patient’s current condition may diminish. Hence, to continue to exploit the information from laboratory test results, we combined NEWS and LDTEWS using a linear decay weight, such that after 120 h (5 days), the score derived from the laboratory information (LDTEWS) is no longer used to determine the risk score, until new laboratory data becomes available. We chose a cut-off of 5 days, as this was the average length of stay for patients on whom the LDTEWS scores was developed [14]. The model is thus a simple linear combination of the two (normalised) scores as a function of time, based on the most recent available laboratory data, as shown in the following equation:

(1) $$\text{LDTEWS}:\text{NEWS}\;\text{Risk}\;\text{Index}=\omega \times \text{LDTEWS}+\left(1-\omega \right)\times \text{NEWS}\;,$$

with

(2) $$\omega =\beta \left(1-\frac{TimeSinceLabs}{120}\right)$$

where TimeSinceLabs corresponds to the time (in hours) since the last *individual* laboratory test result was recorded and has a maximum value of 120 h. For example, if the laboratory has just measured a new haemoglobin result at the same time that vital signs were obtained, but all other blood tests values were measured the previous day, *TimeSinceLabs* would take a value of 0. This was to ensure that acute changes (e.g. a drop in haemoglobin) in laboratory results data were given appropriately increased weighting.

The coefficient β is used to adjust the weight of LDTEWS with respect to the time that the *latest* of each laboratory tests has been received. This approach allows the blood tests to gradually “age-out” as they become too far removed in time to be (potentially) relevant. At a minimum, computing a patient’s risk index is no different from computing NEWS, normalised between 0 and 1; i.e. if TimeSinceLabs ≥120, then ω=0 and Risk Index=NEWS.

We determined the value for the coefficient β using the development cohort. In order to achieve better generalisation, we split the data set randomly into a 10 partitions. In each partition, a grid-search approach (with the following possible values of $\beta =\left[0.01,0.02,0.03,\ldots ,0.99\right]$) was employed and we found the value of β that maximised the discriminative ability of the risk index using the primary outcome, as given by the area under the receiver-operating characteristics curve (AUROC) or c-statistics. The final coefficient β was selected as that corresponding to the maximal mean AUROC across all cross-validation folds. The approach is equivalent to what is commonly referred to as “cross-validation”.

We assessed performance of the risk index using the two validation sets. The c-statistic represents how well the scoring system discriminates observation sets followed by an adverse outcome (in the case of the primary outcome, in-hospital death or unanticipated ICU admission) from those with no subsequent adverse outcome within the next 24 h. Calibration is assessed visually by comparing the observed risks of the primary outcome against each possible value of the risk index.

We compared the performance of the risk index with that of NEWS, and a variant of the combined NEWS and LDTEWS score in which the weight ω is set to be fixed at ω=β (i.e., without the linear decay).

All analyses were performed using the R statistical software (v3.4.4) [23] and ROC curves were calculated using the pROC package [24].

## Results

### Participants and demographics

**Fig. 1:**
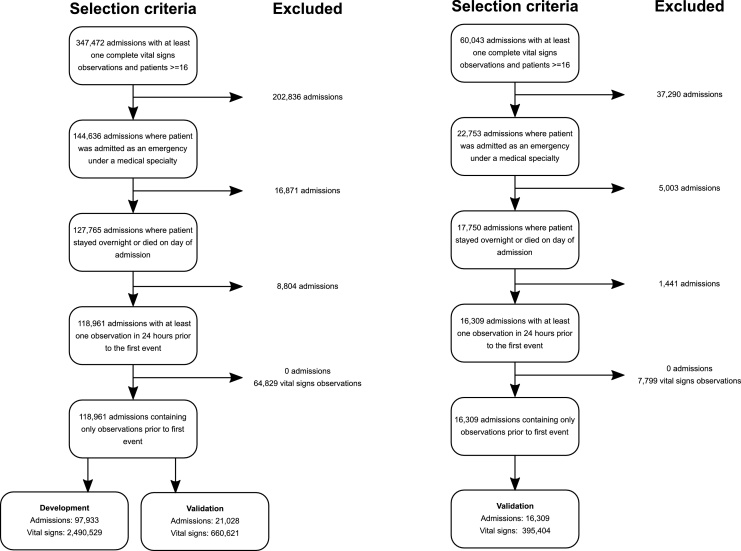
Diagram showing construction of development and validation sets in Portsmouth and Oxford.

Fig. 1 summarises the application of exclusion criteria to patient admissions in order to derive the development and validation cohorts in both organisations. Table 1 provides a summary of admission characteristics of the development and validation cohorts derived from emergency admissions to selected medical specialties in both organisations. The development cohort had a total of 97, 933 admissions containing 2,490,529 unique vital signs observations, with a median age of 73 (IQR 57–83) and median Charlson co-morbidity index of 4 (0–13). In this cohort, 4.1% of admissions resulted in death in hospital, with 1.1% of admissions transferred to an ICU from the wards. Validation cohorts from Portsmouth and OUH were similar (Table 2).

**Table 2:** Demographics of admissions for development and validation cohorts.

	Portsmouth Development	Portsmouth Validation	Oxford Validation
Dates	01/2011–12/2015	01/2016–12/2016	01/2016–12/2016
Admissions, n	97,933	21,028	16,309
Complete vital signs sets, n	2,490,529	660,621	395,404
Unique laboratory results, n	458,397	110,074	62,089
Mortality, n (%)	4723 (4.8%)	1031 (4.9%)	721 (4.4%)
ICU admissions, n (%)	1078 (1.1%)	216 (1.0%)	159 (1%)
Males, n (%)	48,189 (49.2%)	10,366 (49.3%)	8124 (49.8%)
Age, Median (IQR)	73 (57–83)	74 (59–84)	73 (58–84)
Charlson co-morbidity index, Median (IQR)^a^	4 (0–13)	4 (0–13)	4 (0–14)
Length of stay (days), Median (IRQ)	3.7 (1.5–8.8)	4.0 (1.8–10.2)	3.5 (1.4–8.2)

a The Charlson Comorbidity Index was determined according to the methodology and specification provided by NHS Digital (available at https://beta.digital.nhs.uk/publications/ci-hub/summary-hospital-level-mortality-indicator-shmi).

### Risk index development and specification

During the development phase, a value of β=0.26, for linear time-decay weighting function (see Eq. (2)) was obtained and used to calculate the risk index (Eq. (1)).

### Validation of the risk index

**Fig. 2:**
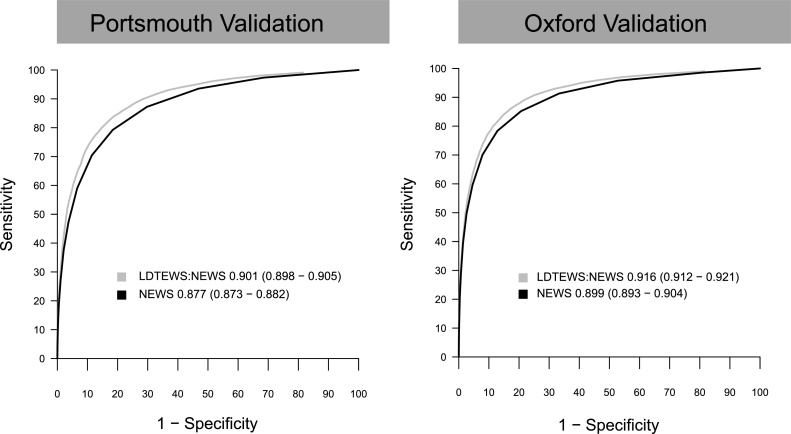
ROC curves showing the performance of NEWS, LDTEWS and the new combined score to predict observation sets followed by in-hospital death or unanticipated admission to ICU in the following 24 h.

Fig. 2. ROC curves showing the performance of NEWS, LDTEWS and the new combined score to predict observation sets followed by in-hospital death or unanticipated admission to ICU in the following 24 h are plotted in Fig. 2. Receiver Operator Curves (ROC) show the balance of sensitivity and specificity across the range of NEWS, and the risk index (denoted LDTEWS:NEWS) in each of the validation cohorts for predicting unanticipated admission to ICU or in-hospital death within 24 h. The combined LDTEWS:NEWS score consistently shows superior discrimination to NEWS alone. In Portsmouth, LDTEWS:NEWS gave a c-statistic of 0.901(95% CI 0.898-0.905) versus 0.877(0.873-0.882) for NEWS; in the OUH organisations the results were similar, in that the performance of the combined score was higher, 0.916 (0.912-0.921), than that for NEWS, 0.899 (0.893-0.904). When compared to standard NEWS trigger levels (5 and 7), the LDTEWS:NEWS thresholds (0.27, 0.36) with comparable specificity both showed higher positive predictive value (Supplementary Table 4).

## Discussion

### Main findings and strengths

We have developed a novel risk index by combining two previously published early warning scores, which can be calculated at any given time using patients’ most recent vital signs and laboratory test results. Our study shows that commonly measured laboratory tests collected throughout the hospital stay, represented by a simple EWS (LDT-EWS), can be combined with NEWS to increase the ability to identify patients at risk of subsequent ICU admission or in-hospital death within 24 h. We validated these findings using data from a different hospital trust, as well as in a validation cohort from the development site.

The results obtained in this study are consistent with previous studies [15,16,25,26], where incorporating additional clinical data (e.g. blood tests) with physiological measures (e.g. vital signs) improves the ability of models to predict those patients at risk of adverse outcomes. However, while others have used more complex machine learning approaches, such as neural networks [16,27], our study shows how a simple and easily interpretable score can still increase performance above that of NEWS alone. We demonstrate how existing early warning scores, which rely on the use of different inputs acquired at different times, can be blended together using a simple linear combination of their outputs. Moreover, our risk index has consistent performance improvement in two different UK organisations, with respect to the NEWS system.

### Limitations

We excluded elective admissions and patients admitted to surgical specialties from this study in order to increase homogeneity of patient cohorts in each hospital trust. Although laboratory results obtained in the emergency department were included, vital signs measured in this department were unavailable. Although the validation cohorts in each of the two organisations reflect patient admissions over the same year, we were unable to adjust for all potential confounders, such as the different case-mix in the two hospital groups. In addition, we were unable explicitly to identify patients on end-of-life pathways, so we instead excluded patients who had no observations in the 24 h prior to discharge as a proxy.

It is recognised that not all laboratory test results included in LDT-EWS have identical durations of relevance [25]. Therefore, a more sophisticated treatment of the laboratory test inputs, which allows for different decay-functions and phase-out periods for each individual test (or LDT-EWS component), may enhance the performance of models combining laboratory test results and NEWS. In addition, our risk index does not consider temporal trends in laboratory tests or vital signs, or patients’ baseline (e.g. an acute versus chronic renal impairment). We are currently exploring methods to incorporate these trends into the risk index.

We also note the overall contribution of NEWS to the risk index is substantially different to that of LDT-EWS. The contribution of both early warning scores in computing the risk index can be estimated from the maximum and minimum possible values of the weighting function (Eq. (2)). Hence, the contribution of LDT-EWS to the risk index varies from 0 to 26%, and the contribution of NEWS varies from 74 to 100% (note that both scores were normalised to have the same scale from 0 to 1). This could be due to a number of factors, such as the known lag between the time the blood sample is collected and the time the results are available in the electronic health record system. As we recalculate the risk index at the time of a vital signs observation, it is reasonable to assume that the vital signs will be more directly associated with the *current* physiological status of the patient than the results of a blood sample collected a few hours before. In addition, while NEWS was originally designed to predict in-hospital mortality within 24 h [3,22], LDT-EWS was originally designed to predict in-hospital mortality (at any time during the hospital stay) [14], which might contribute to its lower relevance for discriminating short-term outcomes.

## Ethical approval and trial registration

Health Research Authority approval was obtained for gathering the data used in this study from the Research Ethics Committee (REC reference: 16/SC/0264). Additional ethics approval for Portsmouth 08/02/1394.

(https://www.hra.nhs.uk/planning-and-improving-research/application-summaries/research-summaries/haven/)

## Conflicts of interest statement

VitalPAC™, the system used to collect vital signs data in Portsmouth, is a collaborative development of The Learning Clinic Ltd (TLC) and Portsmouth Hospitals NHS Trust (PHT). At the time of the research, PHT had a royalty agreement with TLC to pay for the use of PHT intellectual property within the VitalPAC™ product. PS is employed by PHT. GS was an employee of PHT until 31/03/2011. DP was an employee of PHT until 31/07/2016. Until October 2015, PS and the wives of GS and DP were minority shareholders in TLC. GS is a member of the Royal College of Physicians of London’s National Early Warning Score (NEWS) Development and Implementation Group (NEWSDIG), which developed NEWS. DP assisted the Royal College of Physicians of London in the analysis of data validating NEWS. PW and LT co-developed the System for Electronic Notification and Documentation (SEND), for which Drayson Health has purchased a sole licence. The company has a research agreement with the University of Oxford and royalty agreements with Oxford University Hospitals NHS Trust and the University of Oxford. Drayson Health have paid LT consultancy fees as a member of its Strategic Advisory Board and may in the future pay PW personal fees. DC has been recently appointed Research Director of Drayson Health and will in future receive consultancy fees for this role.

## Funding

This publication presents independent research commissioned by the Health Innovation Challenge Fund (HICF-R9-524; WT-103703/Z/14/Z), a parallel funding partnership between the Department of Health and Wellcome Trust. The views expressed in this publication are those of the authors and not necessarily those of the Department of Health or Wellcome Trust. PW is supported by the National Institute for Health Research, Biomedical Research Centre, Oxford.
