# Supplementary material for: Predicting in-hospital mortality and unanticipated admissions to the intensive care unit using routinely collected blood tests and vital signs: Development and validation of a multivariable model
Source: Resuscitation. 2018 Dec;133:75–81. doi: 10.1016/j.resuscitation.2018.09.021 (PMC6562198; doi:10.1016/j.resuscitation.2018.09.021)
Supplement: Supplementary file 1 [file mmc1.docx]

## Supplementary Figures and Tables

### Appendix 1

Supplementary Table 1 shows a comparison of the predictors used in the development and two validation sets. It can be seen that these values are generally comparable between each cohort.

|  | Development | PH Validation | OH  Validation |
| --- | --- | --- | --- |
| Albumin (g/L) | 29 (24-34) | 28 (23-32) | 28 (23-33) |
| AVPU  % A  % V, P or U | 98.77%  1.23% | 99.363%  0.637% | 97.96%  2.04% |
| Creatinine (mmol/L) | 87 (64-140) | 85 (63-135) | 84 (63-125) |
| Haemoglobin (g/L) | 113 (98-128) | 113 (98-128) | 112 (95-129) |
| Heart rate (bpm) | 79 (69-91) | 79 (69-89) | 81 (70-93) |
| Potassium (mmol/L) | 4.1 (3.8-4.5) | 4.1 (3.8-4.5) | 4 (3.6-4.4) |
| NEWS | 1 (0-3) | 1 (0-3) | 2 (1-3) |
| On supplementary oxygen | 19.8% | 21.4% | 17.2% |
| Respiratory rate (bpm) | 17 (16-18) | 17 (16-18) | 18 (17-18) |
| Systolic blood pressure (mmHg) | 124 (110-140) | 123 (110-139) | 125 (110-142) |
| Sodium (mmol/L) | 136 (133-139) | 136 (133-139) | 137 (134-140) |
| Oxygen saturation (%) | 96 (95-98) | 96 (95-97) | 96 (94-98) |
| Temperature (ºC) | 36.7 (36.4-37) | 36.7 (36.4-36.9) | 36.3 (36-36.7) |
| Urea (mmol/L) | 6.9 (4.5-11.8) | 7.3 (4.9-11.9) | 6.7 (4.4-11) |
| White blood cells (10^9^ cells/L) | 8.8 (6.7-11.8) | 8.6 (6.5-11.6) | 8.9 (6.5-12.1) |

Supplementary Table 1 Median and interquartile ranges for all 14 predictors (plus NEWS score) in the development and two validation cohorts

|  | PH Validation | | OH Validation | |
| --- | --- | --- | --- | --- |
| Score | *Death* | *UICU* | *DEATH* | *UICU* |
| NEWS | 0.887  (0.882 - 0.892) | 0.838  (0.827 - 0.849) | 0.905  (0.899 - 0.911) | 0.876  (0.865 - 0.887) |
| NEWS:LDTEWS | 0.911  (0.907 - 0.915) | 0.862  (0.852 - 0.872) | 0.923  (0.917 - 0.928) | 0.894  (0.884 - 0.903) |

Supplementary Table 2 C-statistic with 95% confidence intervals for the individual secondary outcomes (death, unanticipated ICU admission as the first event) within 24 hours of an observation set


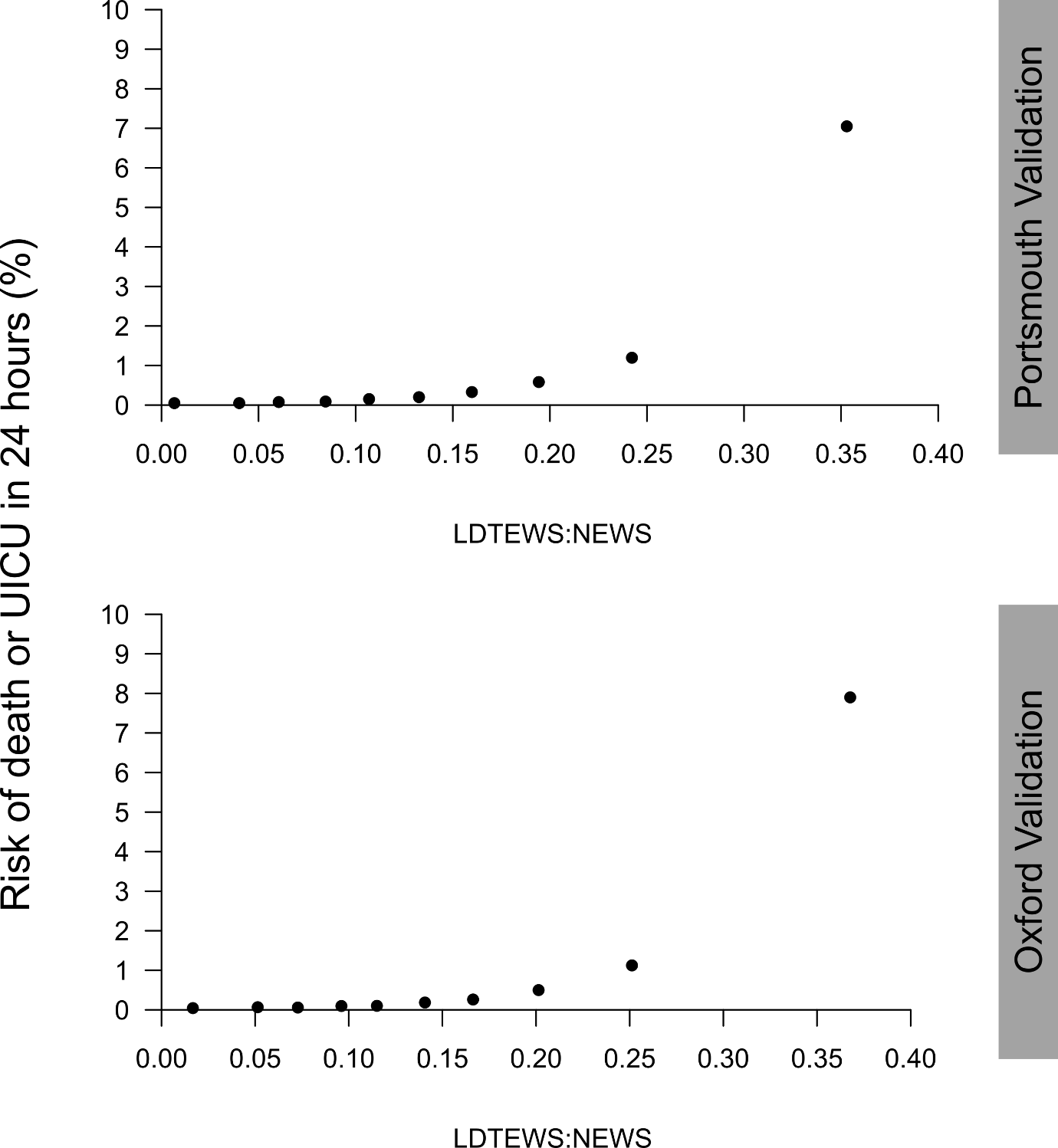


Supplementary Figure 1 Relationship between LDTEWS:NEWS score and risk of death or unanticipated ICU admissions within 24 hours of a vital-sign observation set. The x-axis shows the LDTEWS:NEWS score and the y-axis shows the risk of death or unanticipated ICU admissions within 24 hours of a vital-sign observation set (given as a percentage)


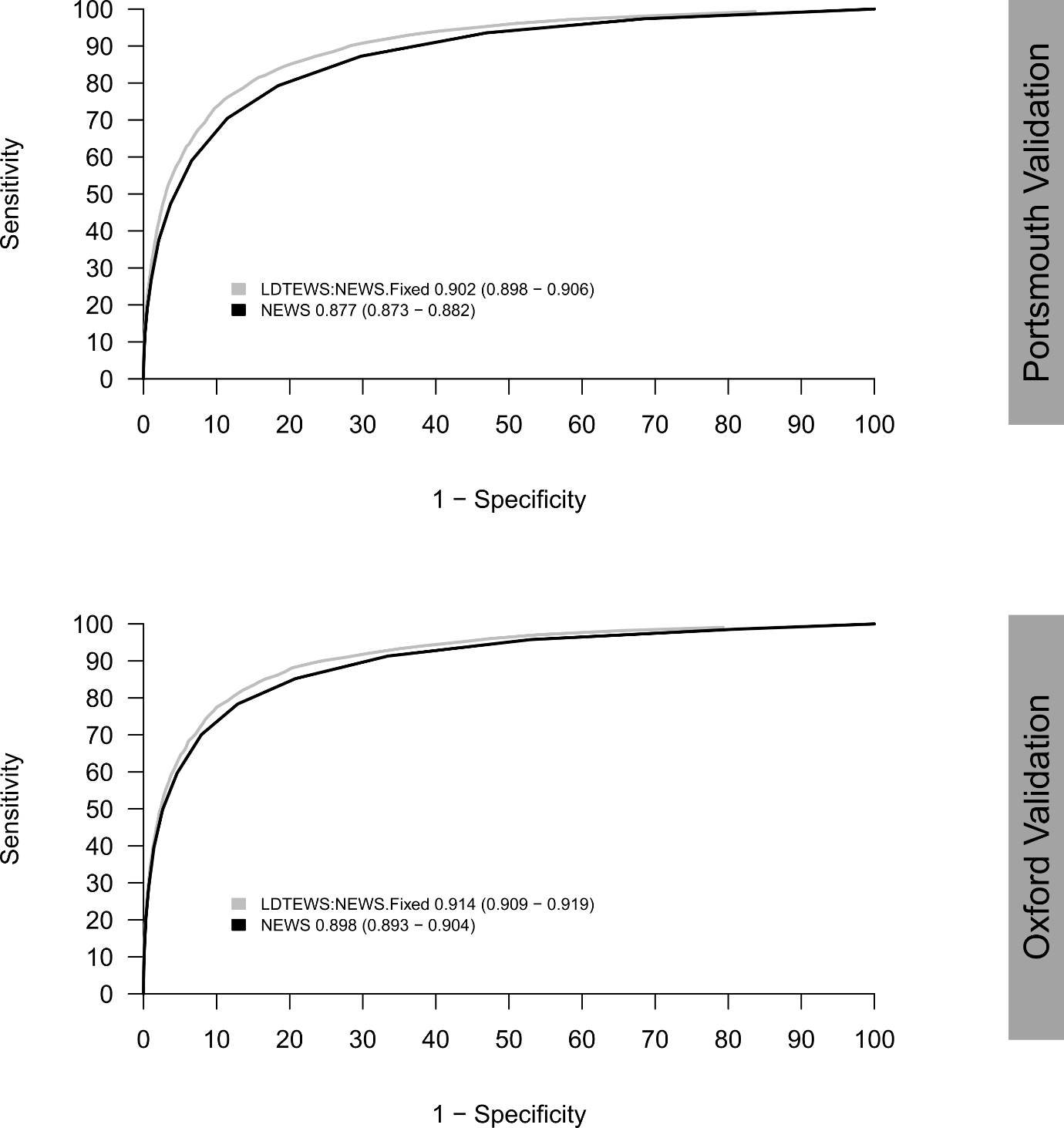


Supplementary Figure 2 ROC curves showing the performance of NEWS, LDTEWS and a combined score (LDTEWS:NEWS.Fixed) to predict which vital-sign observation sets will be followed by in-hospital death or unanticipated admission to ICU in the following 24 hours. Unlike LDTEWS:NEWS, LDTEWS:NEWS.Fixed has fixed relative weighting (0.25) regardless of how recently the laboratory tests have been performed (up to 5 days, where the weighting is fixed at 0)

For the recommended NEWS trigger thresholds (5 and 7), we sought an equivalent threshold for LDTEWS:NEWS that was at least as specific for the primary outcome (Supplementary Table 3). It can be seen that the sensitivity and positive predictive value (PPV) are higher for LDTEWS:NEWS (with trigger thresholds of 0.27 and 0.36) in both the PH and OUH validation cohorts.

|  | PH Validation | | | OUH Validation | | |
| --- | --- | --- | --- | --- | --- | --- |
| Score/Threshold | *Sens* | *Spec* | *PPV* | *Sens* | *Spec* | *PPV* |
| NEWS ≥5 | 70.4% | 88.5% | 5.73% | 78.4% | 87.1% | 5.97% |
| <NEWS:LDTEWS> ≥ 0.27 | 75.9% | 88.9% | 7.00% | 80.5% | 88.0% | 6.57% |
| NEWS ≥7 | 47.3% | 96.3% | 11.2% | 59.6% | 95.4% | 11.9% |
| NEWS:LDTEWS ≥0.36 | 53.9% | 96.5% | 14.6% | 59.6% | 96.2% | 14.0% |

Supplementary Table 3 Sensitivity, specificity and positive predictive value (PPV) at standard NEWS trigger thresholds (5 and 7) and at least equally specific thresholds for LDTEWS:NEWS (0.27 and 0.36).

### Appendix 2

In each validation cohort, we also evaluated the performance of NEWS and the LDTEWS:NEWS by repeatedly sampling (10,000 times) one observation set per admission by selecting the observation closest to a random time within the admission (see for a detailed description of the method [28]). A c-statistic was calculated for each sample – Supplementary Table 2 below shows the mean and 95% confidence intervals for each evaluation.

| Score | PH Validation | OH Validation |
| --- | --- | --- |
| NEWS | 0.887 (0.874-0.899) | 0.912 (0.899-0.925) |
| NEWS:LDTEWS | 0.912 (0.902-0.923) | 0.930 (0.918-0.94) |

Supplementary Table 4 C-statistic with 95% confidence intervals taking a random observation per admission using the primary outcome (death or unanticipated ICU admission as the first event within 24 hours of an observation set)
